# Copper tungstate-assisted photocatalytic degradation of industrial products (dyes and pharmaceuticals) in water

**DOI:** 10.1039/d5ra09421f

**Published:** 2026-04-17

**Authors:** Dhanalakshmi Vadivel, Jessica García, Nithishkumar Kameswaran, Daniele Dondi

**Affiliations:** a Laboratory of Radiation and EPR Spectroscopy, Department of General Chemistry, University of Pavia Via Torquato Taramelli 12 27100 Pavia Italy dhanalakshmi.vadivel@unipv.it

## Abstract

The contamination of ecosystems, specifically aquatic ecosystems, has emerged as a substantial concern in recent decades. This is mostly owing to the extensive growth of large industries that not only promote societal advancements but also impose adverse effects on the environment. Azure A (AA) and Azure B (AB) are the cationic dyes commonly employed in industrial and biomedical fields as intermediates in the production of several pharmaceuticals and as mediators for electrochemical biosensing, and indigo carmine (IC) is an anionic dye used in the textile industry for dyeing. Micropollutants such as pharmaceuticals, propranolol hydrochloride (β-blocker) (PPH) are the pollutants in the subject of discussion. In this research, the efficient degradation of AA (91%), AB (84.6%), IC (87.3%) and PPH (>80%) by the CuWO_4_ photocatalyst is highlighted. Both the dye and drug degradations followed pseudo-first order kinetics. CuWO_4_ catalyst is used to alleviate the impact of the environment on its ecosystem as a photocatalyst with ultraviolet (UV) irradiation of pollutants (AA, AB, and PPH). For the analysis of pollutant decomposition, UV-visible absorption spectroscopy and high-performance liquid chromatography (HPLC) are employed. This study highlights the potential of nanomaterial-based photocatalysis as a viable and effective method for sustainably mitigating water pollution.

## Abstract 2

The contamination of ecosystems, specifically aquatic ecosystems, has emerged as a substantial concern in recent decades.

## Graphical abstract

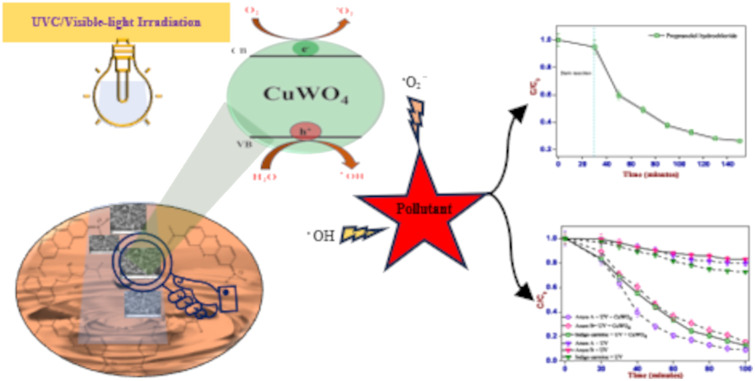

## 1. Introduction

Over the decades, the development of industrialization has gained strength in society; nevertheless, it has also caused negative environmental impacts on ecosystems.^1^ As a report, about 15% of dyes are unscientifically discarded in the environment, especially in aquatic environments, which poses risks for both nature and health.^2,3^ Numerous dyes, including methylene blue (a phenothiazine dye)^4^ and indigo carmine, among others, contain aromatic rings; therefore, their biodegradation is not feasible.^5,6^

“Emerging contaminants” are detected increasingly as low amounts in an ecosystem. The drugs,^7–9^ such as metoprolol, propranolol, atenolol, and pindolol belong to this group of contaminants are fall in this category. These drugs possess characteristic multi-functionalized aromatic groups, and they are soluble in water and susceptible to deionization.^10–12^ In European wastewater, an average concentration of 0.01 µg L^−1^ has been quantified up to concentrations of 0.29 µg L^−1^ for PPH.^13^ Many of the drugs are designed to be consumed orally, as they are resistant to neutral and/or basic hydrolysis, oxidation, or photolysis, which become their main routes of abiotic dissipation in natural waters. In relation to this, propranolol has a naphthalene skeleton, which indicates that it can act as a photosensitizing agent and be unstable to light.^14^

Propranolol hydrochloride (PPH) is a non-selective β-blocker drug used in clinical practice, which is administered mainly during cardiovascular therapy and for the management of primary hypertension.^15–17^ The assertion that the decrease in blood pressure is a consequence of diminished cardiac output caused by the inhibition of beta-receptors in the heart is subject to criticism.^18^ The presence of PPH in water is of concern due to its potential adverse effects on aquatic organisms and aquatic ecosystems in general.^19^ Although PPH is a commonly prescribed beta-blocker, it can be considered safe for humans^20^ only when used correctly. PPH can have adverse effects on aquatic organisms, especially at concentrations higher than the permissible limit in drinking water.

Industrial dyeing generates effluents, which comprise organic contaminants, and require treatment prior to discharge into natural water sources. Contemporary methods for treating wastewater involve the utilization of specialized oxidation techniques, including ozonation, Fenton reactions, and photocatalysis.^21–24^ Considerable interest has been directed towards photocatalysis due to its potential to eliminate toxic inorganic compounds, heavy metals, and organic pollutants from wastewater in a sustainable, eco-friendly, and straightforward manner.^25–27^

Solutions to technological and environmental challenges in the fields of solar energy conversion, catalysis, medicine, and water remediation are offered by nanomaterials.^28,29^ Researchers utilize metal oxide nanomaterials as a catalyst, including those of Fe_3_O_4_, TiO_2_, Al_2_O_3_, CuO, ZnO, and MgO, due to their versatile physical, chemical, and morphological properties that can be customized by tuning their synthesis parameters.^30–33^ The photodegradation of pollutants in water is a process that involve the production of reactive oxygen species.^31^

Conventional methods such as reverse osmosis, hydrogen peroxide treatment, dialysis, and UV photocatalysis^34^ are used for pollutant remediation from water. UV photocatalysis is a highly effective method owing to its easy operation, simple design, and the ability to completely degrade wastewater. The nanomaterials of metal oxides have the ability to degrade dyes present in industrial effluents. Notably, under ultraviolet light, low molecular weight metal oxide nanomaterials can engage in oxidation processes, yielding industrial CO_2_, H_2_O and aliphatic acids as final products. Researchers have shown significant interest in creating inorganic nanomaterials, such as metal oxide, metal tungstate, and metal molybdates, through synthesis.^34,35^

Copper tungstate (CuWO_4_) belongs to the tungstate family of compounds containing divalent transition metal ions that are structurally related. The 3d-orbitals of these metal ions are responsible for the observed electronic correlation effects.^35^ This semiconductor material with a high catalytic capacity is applied for nitric oxide gas sensing^36,37^ and photoelectrochemical water splitting.^38–40^ CuWO_4_ contains Cu^2+^ ions and has a d^9^ electronic configuration unlike common d^0^ photocatalysts such as WO_3_, TiO_2_, and BiVO_4_, and this distinct electronic structure results in different optical transitions, altered charge‑carrier behavior, stronger electronic correlation effects, and consequently unique photocatalytic properties.^41–44^ Recent research indicates that CuWO_4_ nanoparticles exhibit higher photocatalytic activity than P25.^35^ CuWO_4_ is formed through the reaction of Cu^2+^ with WO_3_ at high temperatures,^45^ and it can be produced in the form of thin films *via* the controlled addition of Cu^2+^ on the WO_3_ substrate.^46^

The present study aims to understand the degradation of PPH, AA, AB, and IC by the green photocatalyst^42^ CuWO_4_ under UVC and visible light irradiations. Analytical techniques such as UV-visible absorption spectroscopy and HPLC are used for the performance evaluation of CuWO_4_, and kinetic studies are also performed. The heterogeneous nature of the catalyst facilitates its subsequent removal following its utilization.^47^

## 2. Experimental methods

### 2.1. Chemicals

**Scheme 1:**
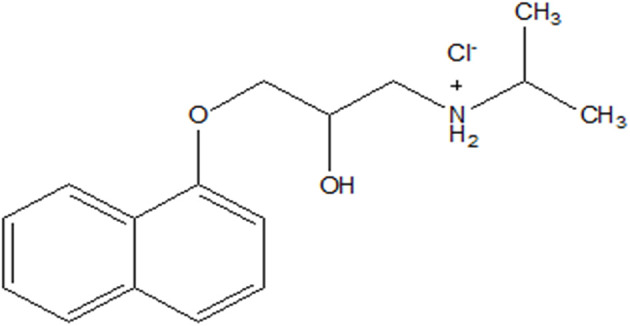
Structure of propranolol hydrochloride.

The compounds copper(ii) sulphate pentahydrate CuSO_4_·5H_2_O (≥98%), AA, AB and IC were acquired from Sigma-Aldrich, USA. Sodium tungstate dihydrate, Na_2_WO_4_·2H_2_O (≥99%), was acquired from Sigma-Aldrich, India. dl-propranolol hydrochloride (99%) (Scheme 1) was provided by Thermo Fisher Scientific, Japan. Acetonitrile (ACN) for HPLC gradient grade (≥99.9%), phosphoric acid (H_3_PO_4_), and water for HPLC (H_2_O) were obtained from Sigma-Aldrich, France. For the synthesis of the catalyst, AA, and AB, we used double-distilled water. Nevertheless, to spike the IC, we used tap water from Pavia (Italy).

### 2.2. Synthesis of CuWO_4_

CuWO_4_ was prepared *via* coprecipitation by mixing two aqueous solutions: 50 mL of 0.4 M solution A (CuSO_4_·5H_2_O) and 50 mL of 0.4 M solution B (Na_2_WO_4_·2H_2_O). Solution B was poured into solution A under stirring at a temperature of 60 °C and kept for 2 hours; subsequently, it was filtered, and the solid was dried at 100 °C for 24 hours in an oven, obtaining copper tungstate dihydrate.^48^

### 2.3. Instrumentation for degradation analyses

**Scheme 2:**
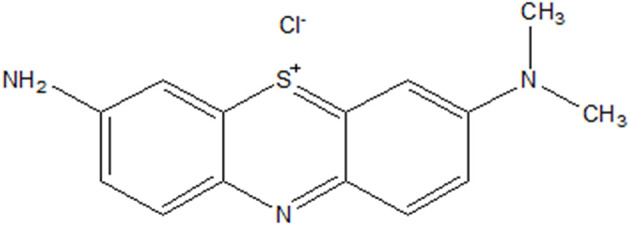
Structure of azure A.

**Scheme 3:**
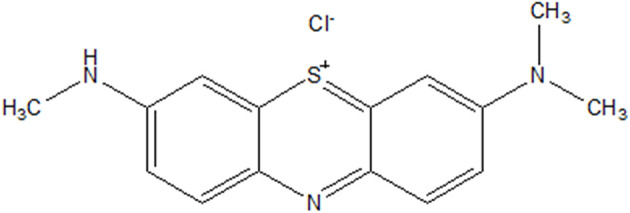
Structure of azure B.

AA (Scheme 2), AB (Scheme 3) and IC dye decomposition analyses were performed by UV-visible absorption spectroscopy (Hewlett Packard 8452A diode array spectrophotometer) in 1 cm quartz cuvettes. The drug, PPH, was analyzed by HPLC. The HPLC setup is as follows: The Waters' HPLC system has an HPLC-UV-vis detector WATERS 2487 set to 291 nm. The system was equipped with a C-18 reverse phase Phenomenex column (150 × 4.6 mm, particle size 5 µL). The operating parameters for the HPLC analysis are as follows: a mobile phase consisting of a mixture of acetonitrile (ACN, 30% by volume) and water containing 0.1% of phosphoric acid (H_3_PO_4_) (70% by volume), a column temperature of 30 °C, an injection volume of 10 µL, and mobile phase flow rate set at 1 mL min^−1^.

### 2.4. Sample preparation

**Scheme 4:**
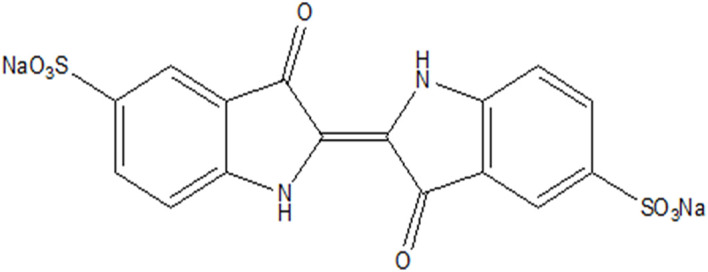
Structure of indigo carmine.

A suspension of 50 ppm of propranolol hydrochloride, was added to 50 mg of CuWO_4_ in 100 mL. In the case of dye decomposition, 5 mg of CuWO_4_ was used in 25 mL of dye solution with a concentration of 5 × 10^−5^ mol dm^−3^. As the samples (dyes or/and drugs) were adsorbed on the CuWO_4_, the photocatalytic activity of the nanoparticles was tested. The dye samples were kept in darkness for 20 minutes. Then they were irradiated in quartz tubes inserted in a photoreactor equipped with 4 low pressure mercury lamps of 15 W each. All these solutions were also verified (maximum irradiation time of 2 h) by irradiating with an 18 W visible light LED lamp at 30 mW cm^−2^ (EvoluChem P204-18-1 6200K LED0000034) which has no appreciable reactivity. In the case of PPH this visible light is sufficient to perform the photo reaction. In the case of IC, we employed the dye in tap water to understand its decomposition efficiency as industrial waste on a real time scale (Scheme 4).

## 3. Results and discussion

### 3.1. Characterization of CuWO_4_

#### 3.1.1. X-ray diffraction

**Fig. 1:**
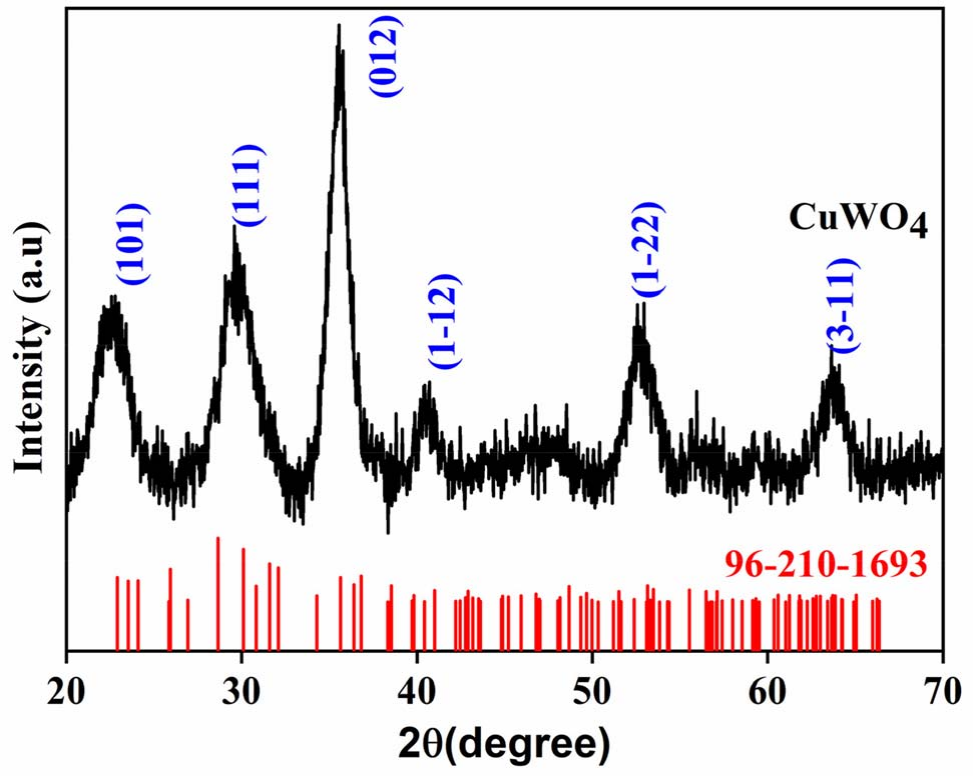
X-ray diffraction pattern of CuWO_4_.

Fig. 1 illustrates the X-ray diffraction pattern of the prepared CuWO_4_, which is in agreement with triclinic CuWO_4_ that belongs to the *P*1̄ space group in the open crystallographic database 2024 (JCPDS number: 96-210-1693). The obtained diffraction patterns matched with the (101), (111), (012), (1−12), (1−22) and (3−11) planes of triclinic CuWO_4_.

#### 3.1.2. UV-visible absorption spectrum of CuWO_4_ and the Tauc plot of CuWO_4_ for the bandgap calculation

**Fig. 2:**
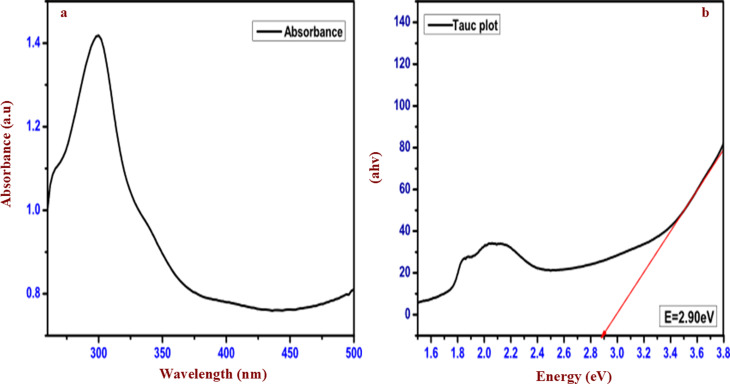
(a) UV-visible absorption spectrum of CuWO_4_. (b) Tauc plot of CuWO_4_.

Fig. 2a depicts the UV-visible absorption spectrum of the as-prepared CuWO_4_ photocatalyst, which displays the absorption maximum at around 300 nm. Fig. 2b displays the Tauc plot of CuWO_4_, which reveals that the optical bandgap energy is 2.90 eV. The obtained bandgap value of the CuWO_4_ is consistent with the past investigation of CuWO_4_, which is 2.25 eV to 2.4 eV.^49–51^ This narrow bandgap of CuWO_4_ enables it to resist photocorrosion^39,52^ in aqueous solutions at a neutral pH. In this context, we tried CuWO_4_ as a photocatalyst powered by visible light, owing to its capability of capturing visible light at a wavelength of 540 nm.^53,54^

At lower concentrations of the dyes and the drug, the CuWO_4_ photocatalyst performed more efficiently under UVC light. We selected lower concentrations of dyes and drugs because the study focuses on conditions relevant to industrial effluents.

#### 3.1.3. Scanning electron microscopy analysis

**Fig. 3:**
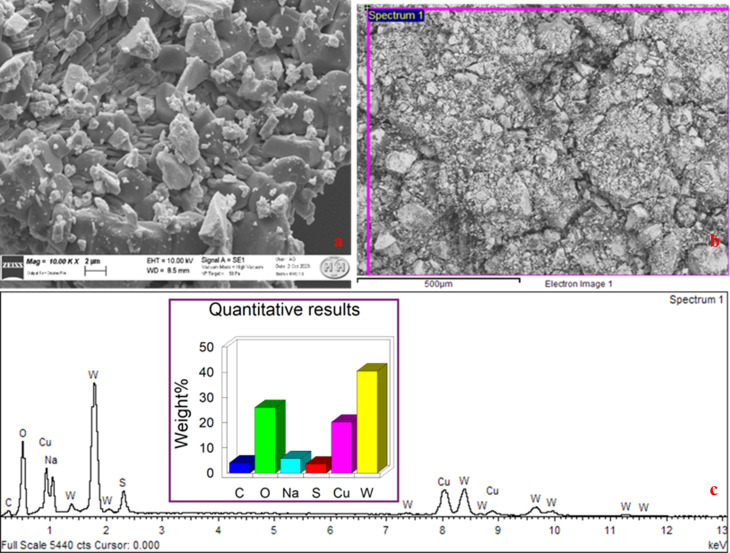
(a and b) Scanning electron microscopic image of CuWO_4_. (c) Elemental distribution of CuWO_4_, showing the presence of (O), copper (Cu), and tungsten (W).

In Fig. 3a and b, the SEM images of CuWO_4_ thin films with a thickness of 2 µm are shown, which reveal irregular granule-like structures above 500 µm. Fig. 3c shows the elemental distribution of CuWO_4_, with Cu and W as the major elements.

The proportional amounts of oxygen (O), copper (Cu), and tungsten (W) obtained from the elemental analysis of CuWO_4_ photocatalyst as measured by SEM/EDX are shown in Table 1. Each of the values given would be acquired from the elemental analysis technique employed and indicate the proportional abundance of each element in the CuWO_4_ photocatalyst as measured by SEM/EDX.

**Table 1:** Percentage of elements present CuWO_4_, as determined by EDX

Elements	Weight percentage (%)	Atomic percentage (%)
Copper (Cu)	17.24	11.07
Tungsten (W)	36.01	7.68
Oxygen (O)	28.05	56.78

#### 3.1.4. Thermogravimetric analysis

Thermogravimetric analysis (TGA) was conducted on a CuWO_4_ sample to figure out its thermal degradation characteristics. TGA was performed under a nitrogen atmosphere (which provides an inert environment) within a temperature range of 25–1000 °C. In the absence of oxygen, the thermochemical processes cause the decomposition of volatile constituents, resulting in the formation of a residue.

**Fig. 4:**
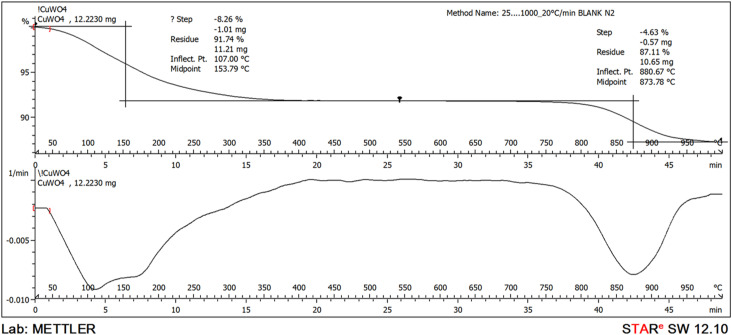
Thermogravimetric analysis (TGA) and differential thermogravimetric analysis (DTGA) plots of CuWO_4_ samples.

Fig. 4 illustrates the process of thermal degradation of the CuWO_4_ sample. At temperatures below 200 °C, the TG measurements indicated a mass loss of less than 10%. This value is around 200–400 °C which depicts the necessity of the heating of CuWO_4_ catalyst after the synthesis above this temperature to obtain the anhydrous salt. However, since we are performing the application of the catalyst in water, the calcination step was not carried out. In addition, at temperatures above 850 °C, TG measurements indicated a mass loss of 12.86% which confirms the formation of CuWO_4_ which is further confirmed by the thermodynamic stability of the tungsten trioxide.^55,56^

### 3.2. Degradation of dyes using CuWO_4_

The utilization of photocatalysts to degrade dyes offers a viable solution for addressing water pollution issues caused by effluents containing dyes from diverse sectors such as textile, pharmaceutical, and chemical manufacturing. The dye molecules in wastewater will be bound to the photocatalyst surface by physical interactions, such as electrostatic attraction or van der Waals forces. This step increases the density of dye molecules that are in close proximity to the photocatalyst, facilitating their easy decomposition. Here, we selected the dye molecules of AA and AB (cationic dyes) and IC (an anionic dye) to be used in conjunction with the photocatalyst, CuWO_4_. In both cases, the photocatalyst exhibited the capability to gather and accumulate dye molecules in its close proximity.

Upon absorbing photons with sufficient energy, photocatalysts produce electron–hole pairs that initiate dye degradation. Concurrently, reactive oxygen species (ROS) are generated on the catalyst surface such as hydroxyl radicals (˙OH) and superoxide ions when electron–hole pairs react with water and oxygen molecules or adsorbed substances.^31,44,57–59^ ROS with strong oxidative potential break chemical bonds and transform dye molecules into fragments. Photocatalysis maintains its effectiveness by converting dye molecules into non-toxic inorganic chemicals such as CO_2_ and H_2_O,^60^ making it a sustainable and environmentally benign method for treating wastewater.

**Fig. 5:**
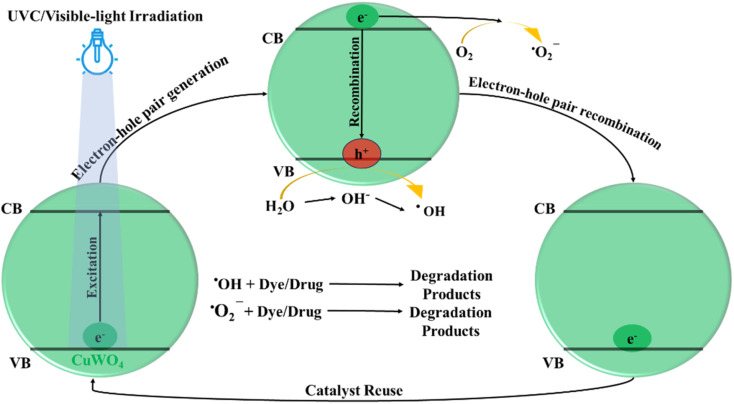
Plausible reaction mechanism for the photocatalytic pollutant degradation using CuWO_4_.

**Fig. 6:**
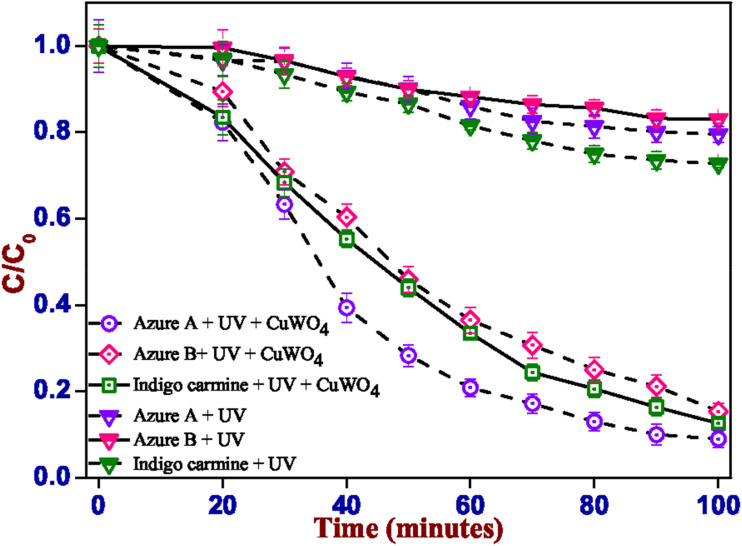
UV-vis absorption spectra of AA, AB, and IC with and without CuWO_4_ under UVC light.

To check the physical interaction (adsorption) of the dyes AA, AB, and IC with CuWO_4_, the catalyst and the solution were stirred for 20 minutes in the dark, as shown in Fig. 6, and the decrease in the absorption value of the solution was measured. It is well known that CuWO_4_ might be adsorbed on the dye surfaces with aromatic ring structures.^61^ After absorbing a photon, CuWO_4_ produces electron–hole pairs. These highly active charge carriers initiate the disintegration of dye molecules. The plausible mechanism reported in the literature is as follows: when the electron–hole pairs react with the oxygen molecules in water or adsorbed compounds, the surface of the photocatalyst forms reactive oxygen species (ROS), such as hydroxyl radicals (˙OH) and superoxide ions (Fig. 5).^31,44,57–59^ The generated ROS have a high oxidation potential, which means that they can break chemical bonds and convert dye molecules into basic chemicals, such as CO_2_ and H_2_O, which are safe for living beings. In the experiment, AA, AB, and IC were subjected to irradiation in the absence of CuWO_4_, as seen in Fig. 6.

**Fig. 7:**
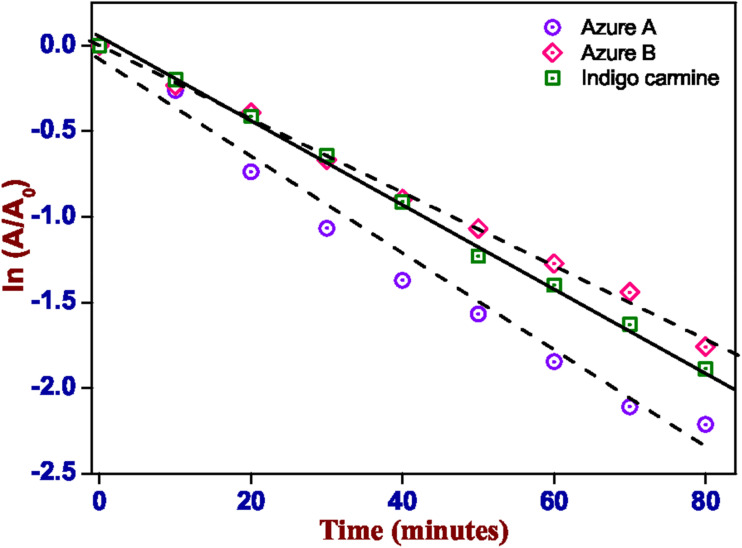
Kinetics of the degradation of dyes (AA, AB, and IC) using CuWO_4_ under UVC irradiation.

The kinetics of the photoreactions were found to follow a pseudo-first-order model (Fig. 7). The kinetic constants were found to be, −2.4 × 10^−2^ a.u. min^−1^ and −2.14 × 10^−2^ a.u. min^−1^ for IC and AB, respectively. For AA, the pseudo-first order kinetic constant is −2.85 × 10^−2^ a.u. min^−1^; however, the deviation from linearity, as visible in Fig. 6, and the low *R*^2^ value (0.98) indicate that the underlying mechanism is probably more complex. The pseudo-first order rate constants were calculated using eqn (1).^62,63^

1 ln(*C*/*C*_0_) = −*kt*

To interpret the photoreaction dynamics, we calculated the amount of light absorbed by the catalyst with respect to dyes. Since the UVC lamp emits mainly at 254 nm, the radiation could be considered monochromatic, and hence, the calculation was performed at this wavelength. The AA, AB and IC dyes used in this experiment exhibited a measured molar absorption of 75 300, 92 600 and 20 500 dm^3^ mol^−1^ cm^−1^, respectively. In these experiments, CuWO_4_ is about 20 times more concentrated, thus drugs have a competitive absorption with the photocatalyst ranging from 50 to 70% of the light absorbed by dyes. Under these conditions, we expect an energy transfer from the excited state of the dye (that we demonstrated to be nonreactive) to the catalyst, giving rise to electron/hole pairs. By fast recombination of electron/hole pairs, molecules adsorbed onto the surface are mainly decomposed. This fact gives importance to the study of adsorption (a dark reaction) before irradiation.

### 3.3. Degradation of PPH by CuWO_4_

**Fig. 8:**
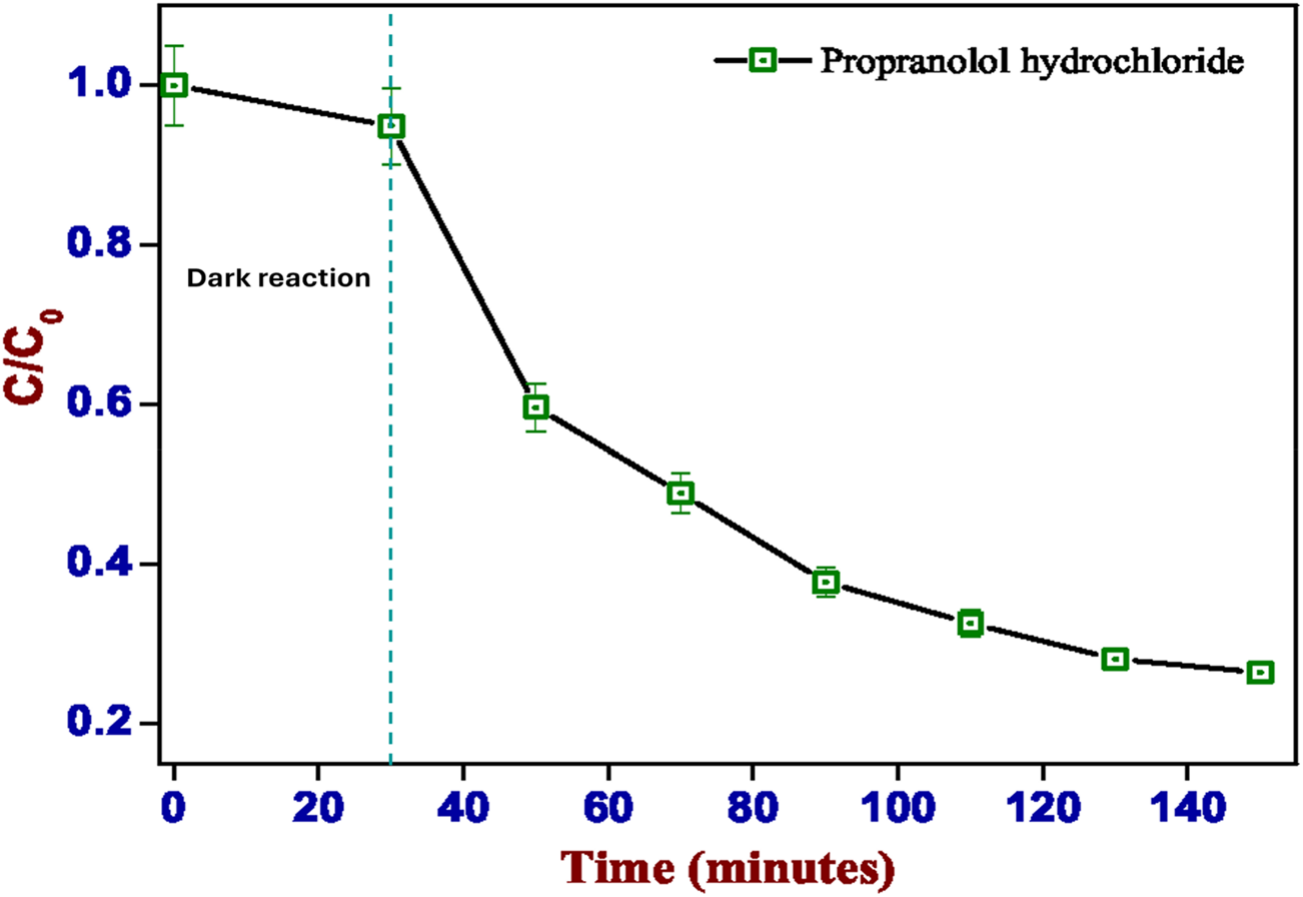
Percentage of PPH degradation in the presence of CuWO_4_ under visible light and in dark conditions.

Fig. 8 summarizes the outcomes of the degradation of PPH under the influence of the CuWO_4_ catalyst. Within 140 minutes, >80% of PPH removal from the solution was achieved. Dark equilibration for 30 minutes revealed only a negligible interaction between the dye and the catalyst. On exposure to visible light, the dye concentrations reduced sharply within the first period of illumination. The kinetics of degradation were later determined using HPLC.

The presence of CuWO_4_ facilitates chemical interactions, creating a conducive environment for the activation of PPH molecules. This activation process involves many transformations, such as oxidation and hydrolysis, which are particularly favored by catalysts of the metal oxide type, such as CuWO_4_.^64^

The addition of CuWO_4_ enhances the degradation efficiency of PPH, resulting in a higher conversion rate of the drug into breakdown products or metabolites. Thus, CuWO_4_ can be advantageous in wastewater treatment applications or in the elimination of medicinal compounds from the environment.^64^

The decomposition of PPH is directly related to the duration of irradiation, whether it is sunlight or artificial light, on the catalyst's surface because irradiation leads to the formation of electron pairs that engage in redox reactions with the adsorbed PPH. The percentage of PPH degradation is subject to variations depending on certain parameters, such as the catalyst type, PPH concentration, reaction conditions, and reaction duration, and modulating these parameters is essential to achieve optimal efficiency in the process.^64–67^

**Fig. 9:**
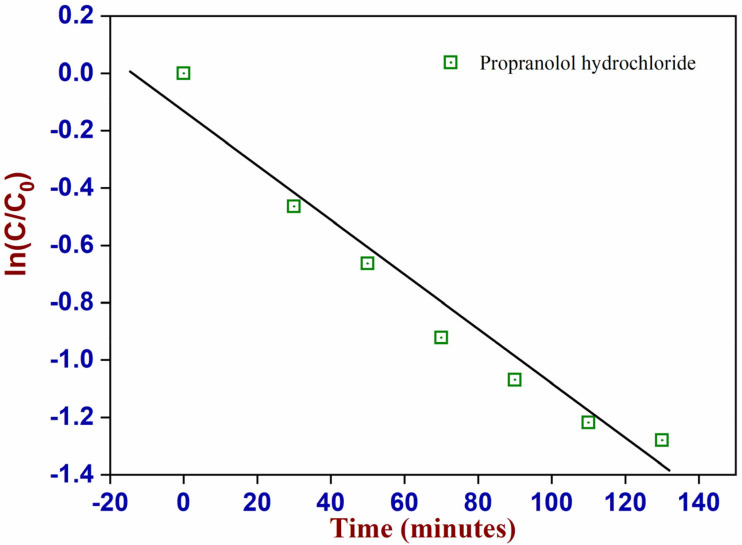
Kinetics of the degradation of PPH using CuWO_4_ under visible light irradiation.

The kinetics of the reaction followed a pseudo-first-order model (Fig. 9). The kinetic constant was calculated to be −0.98 × 10^−2^ a.u. min^−1^ using eqn (1), but the deviation from linearity, as visible in Fig. 7, and the low *R*^2^ value (0.95) indicate that the underlying mechanism is probably more complex. This might be related to the decomposition of PPH by direct light absorption or due to the interactions between secondary products and PPH.

## 4. Conclusion

The effective use of CuWO_4_ (a green photocatalyst) eliminates pollutants such as AA dye (91%), AB dye (84.6%), and IC (87.3%) under UVC irradiation and degrades the drug PPH (>80%) under visible light irradiation. Both the dye and drug degradation reactions follow pseudo-first-order kinetics. Such research on pharmaceutical and dyeing industries will ensure that there is a sustainable and environment-friendly approach to dealing with water pollution. UV-visible absorption spectroscopy and high-performance liquid chromatography (HPLC) are employed to analyze the catalyst's efficacy. These findings indicate the advantages of using material-based photocatalysis (green photocatalyst) as an effective method to mitigate the effects of industrial and medicinal pollutants on the environment. Further exploration and advancements in this domain could potentially result in the development and wider use of enhanced methodologies for water treatment, eventually fostering the preservation of natural environments.

## Conflicts of interest

The authors declare that they have no known competing financial interests or personal relationships that could have influenced the work reported in this paper.

## Data Availability

The data supporting the findings of this study are available from the corresponding author upon reasonable request.
